# Multipotent Adult Progenitor Cells Suppress T Cell Activation in *In Vivo* Models of Homeostatic Proliferation in a Prostaglandin E2-Dependent Manner

**DOI:** 10.3389/fimmu.2018.00645

**Published:** 2018-04-23

**Authors:** Fiona Carty, Jennifer M. Corbett, João Paulo M. C. M. Cunha, James L. Reading, Timothy I. M. Tree, Anthony E. Ting, Samantha R. Stubblefield, Karen English

**Affiliations:** ^1^Department of Biology, Institute of Immunology, Maynooth University, Maynooth, Ireland; ^2^Department of Immunobiology, King’s College London, London, United Kingdom; ^3^Athersys Inc., Cleveland, OH, United States

**Keywords:** multipotent adult progenitor cells, mesenchymal stromal cells, homeostatic proliferation, IL-7, anti-thymocyte globulin, prostaglandin E2

## Abstract

Lymphodepletion strategies are used in the setting of transplantation (including bone marrow, hematopoietic cell, and solid organ) to create space or to prevent allograft rejection and graft versus host disease. Following lymphodepletion, there is an excess of IL-7 available, and T cells that escape depletion respond to this cytokine undergoing accelerated proliferation. Moreover, this environment promotes the skew of T cells to a Th1 pro-inflammatory phenotype. Existing immunosuppressive regimens fail to control this homeostatic proliferative (HP) response, and thus the development of strategies to successfully control HP while sparing T cell reconstitution (providing a functioning immune system) represents a significant unmet need in patients requiring lymphodepletion. Multipotent adult progenitor cells (MAPC^®^) have the capacity to control T cell proliferation and Th1 cytokine production. Herein, this study shows that MAPC cells suppressed anti-thymocyte globulin-induced cytokine production but spared T cell reconstitution in a pre-clinical model of lymphodepletion. Importantly, MAPC cells administered intraperitoneally were efficacious in suppressing interferon-γ production and in promoting the expansion of regulatory T cells in the lymph nodes. MAPC cells administered intraperitoneally accumulated in the omentum but were not present in the spleen suggesting a role for soluble factors. MAPC cells suppressed lymphopenia-induced cytokine production in a prostaglandin E2-dependent manner. This study suggests that MAPC cell therapy may be useful as a novel strategy to target lymphopenia-induced pathogenic T cell responses in lymphodepleted patients.

## Introduction

Despite the significant progress made in solid organ transplantation (SOT) over the past century, allo-immunity remains the greatest barrier to successful graft survival. To avoid allograft rejection, host T cells are depleted and suppressed using induction therapies, followed by long-term maintenance immunosuppressive regimens. Specifically, long-term graft survival (in SOT) is relatively poor and uncontrolled proliferation of T cells in bone marrow transplantation (BMT)/hematopoietic cell transplantation (HCT) in response to lymphodepletion can lead to the development/exacerbation of graft versus host disease (GvHD) (1–4). In the context of BMT or HCT, T cell depletion strategies are used to create space (5). While these therapies have been highly effective at reducing acute rejection and supporting engraftment in the context of BMT or HCT, significant morbidity is associated with homeostatic T cell proliferation that ensues following lymphodepletion (6–10). Furthermore, these immunosuppressive drugs that target T cell proliferation are highly toxic and non-specific, leaving patients on long-term regimens vulnerable to kidney failure, infection, and malignancies (11). Therefore, the development of safer and more effective immunosuppressive regimens is urgently required for transplant patients.

Under normal conditions, T cell proliferation is driven by contact with self-MHC peptide complexes on the surface of antigen-presenting cells and exposure to gamma chain cytokines such as IL-7 and IL-15. Following T cell depletion with induction therapies such as anti-thymocyte globulin (ATG), the availability of IL-7 increases as competition for these stimuli is reduced (6). Thus, peripheral T cells which escape depletion undergo accelerated proliferation. Furthermore, under these conditions, T cells skew toward a Th1 phenotype, while the homeostatic expansion of regulatory cells is slower (7, 12–14). Therefore, lymphopenia-induced proliferation (LIP) induced by lymphodepletion strategies leads to the development of a pro-inflammatory T cell pool promoting allograft rejection (4, 7, 9, 15) and exacerbating GvHD (16, 17).

In order to suppress the homeostatic expansion of T cells following induction therapies and to delay graft rejection, maintenance immunosuppressive drugs such as cyclosporine are commonly employed. While cyclosporine has revolutionized transplantation and undoubtedly prolongs graft survival, as an inhibitor of IL-2 signaling it has a potent inhibitory effect on the expansion of the Treg pool which requires IL-2 to proliferate (18–20). Since Tregs are distinct from other T cells in that they express low levels of IL-7Rα (21, 22), it could be hypothesized that targeting the IL-7 pathway could suppress homeostatic proliferation of conventional T cells without disturbing the expansion of Treg. This hypothesis is supported by the study of Mai et al. (3), whereby administration of an IL-7R antibody following lymphodepletion preferentially inhibited the reconstitution of conventional T cells compared to Treg (3). Furthermore, IL-7 receptor blockade prolongs islet and skin graft survival and prevents GvHD development in *in vivo* models of allo-transplantation (1, 3).

Both mesenchymal stromal cells (MSC) and multipotent adult progenitor cells (MAPC) are bone marrow-derived cell populations that demonstrate pro-reparative and immunomodulatory effects *in vitro* and *in vivo* (23–29). These cell types have shown great promise as facilitators of successful transplantation, as unlike blanket immunosuppressants, MSC and MAPC cells have been shown to suppress pro-inflammatory T cell activity without hindering Treg frequency or activity (29, 30–35). While the exact mechanisms by which MSC and MAPC cells mediate their effects *in vivo* are still unclear, it is known that the vast majority of intravenously injected cells are confined to the lungs before quickly being cleared within a few days (36). Furthermore, MSC and MAPC cells only produce anti-inflammatory factors such as indoleamine 2,3-dioxygenase and prostaglandin E2 (PGE2) upon exposure to pro-inflammatory cytokines (27, 28, 37). Therefore, it is unclear how intravenously administered cells mediate their effects in conditions where inflammation is restricted to distal organs, with some studies showing that local administration is required for therapeutic efficacy in some conditions (31, 32). Recent findings suggest that the retention time of intravenous (IV) administered MSC can be increased through stimulation with cytokines (38). In addition, cytokine pre-stimulation of MSC can influence the distribution of MSC to target organs (38). Moreover, Dazzi and colleagues have demonstrated important differences in the protective effects of apoptotic MSC administered *via* IV versus intraperitoneal (IP) routes (39).

We have previously shown that MAPC cells suppress IL-7-driven stimulation of T cells *in vitro* in a PGE2-dependent manner (28). This study explores the effect and mode of action of MAPC cells on the homeostatic expansion of T cells in two different clinically relevant *in vivo* models of homeostatic T cell proliferation, demonstrating that clinical grade MAPC cells suppress lymphopenia-induced T cell activation *in vivo* in a PGE2-dependent fashion. Furthermore, this study shows that the route by which MAPC cells are administered can affect their therapeutic efficacy in this setting. These findings extend our understanding of MAPC cell-mediated immune suppression and provide important insights for the clinical translation of immune modulatory cellular therapies in the context of conditions requiring lymphodepletion strategies including HCT and SOT.

## Materials and Methods

### MAPC Cell Isolation and Generation

Multipotent adult progenitor cells were isolated from bone marrow of healthy donors by Athersys/ReGenesys as previously described (40). Briefly, human MAPC cells were isolated from a single bone marrow aspirate, obtained with consent from a healthy donor, and cultured in fibronectin-coated plastic tissue culture flasks. Cell cultures were maintained under low oxygen tension in a humidified atmosphere of 5% CO_2_. Cells were cultured to subconfluence in MAPC cell culture media (low-glucose DMEM) (Life Technologies Invitrogen) supplemented with FBS (Atlas), ITS liquid media supplement (Sigma), MCDB (Sigma), platelet-derived growth factor (R&D Systems), epidermal growth factor (R&D Systems), dexamethasone (Sigma), penicillin/streptomycin (Life Technologies Invitrogen), 2-phospho-l-ascorbic acid (Sigma), and linoleic acid–albumin (Sigma). Cells were passaged every 3–4 days and harvested using trypsin/EDTA (Life Technologies Invitrogen). Flow cytometric analysis of surface-expressed antigens confirmed that MAPC cells used in this study were a homogenous population. The cells were positive (>90%) for CD49c and CD90 and negative (<5%) for MHC class II and CD45 (all Abs were from BD Biosciences). Cells were cryopreserved in PLASMA-LYTE A (Baxter) with DMSO and human serum albumin. Prior to *in vivo* administration, MAPC cells were removed from liquid nitrogen and thawed before being washed. MAPC cells were then counted and washed twice in sterile PBS (Sigma-Aldrich). For imaging experiments, freshly thawed MAPC cells were washed in MAPC cell media and incubated at 10 × 10^6^ cells/ml for 1 h with Qtracker^®^ 625 cell label (Life Technologies) followed by two washes in MAPC cell media and three washes in sterile PBS.

### *In Vivo* Model of IL-7-Driven Homeostatic Proliferation

Adult B6.SJL-*Ptprca Pepcb*/BoyJ (C57BL/6-CD45.1) mice (Jackson Laboratories) were used for *in vivo* experiments under the guidelines of the Health Products Regulatory Authority (HPRA) and the approval of the research ethics committee of Maynooth University (under approval number BSRESC-2017-011). The protocol used for IL-7 experiments was adapted from a study by Martin et al. wherein IP administration of recombinant IL-7 conjugated to an IL-7 antibody induced T cell proliferation *in vivo* (41). Thus, 2 µg recombinant murine IL-7 (Peprotech) was incubated with 10 µg of the IL-7 antibody M25 (Biocell) for 30 min at 37°C in PBS. This complex was then administered *via* IP injection on days 0, 2, and 4. Human MAPC cells (1 × 10^6^) in PBS were thawed and administered *via* IP or IV injection on day 1. Mice were sacrificed by cervical dislocation on day 5, and spleens and lymph nodes were harvested for processing.

### *In Vivo* Model of Lymphopenia-Driven Homeostatic Proliferation

Adult B6.SJL-*Ptprca Pepcb*/BoyJ (C57BL/6-CD45.1) mice were used for *in vivo* experiments under the guidelines of the HPRA and the approval of the research ethics committee of Maynooth University (under approval number BSRESC-2017-011). 50 mg/kg ATG or control serum (Accurate Chemical, New York, NY, USA) was administered IP on days 0 and 3. Human MAPC cells (1 × 10^6^) were thawed and administered either IP or IV on day 4. Mice were sacrificed by cervical dislocation on day 7, and spleens and lymph nodes were harvested for processing. In experiments where indomethacin was introduced, 30 µg indomethacin (Sigma-Aldrich) was administered IP to the appropriate groups on days 4, 5, and 6.

### Tissue Processing

Spleens and mesenteric, inguinal, and axillary lymph nodes were harvested, and a single cell suspension was prepared by dissociating the organs and passing through a 40 µm pore cell strainer. Spleens and lymph nodes were centrifuged at 350RCF, and splenocytes were treated with 1 ml 1× red blood cell lysis buffer (Biolegend) for 2 min before being quenched with complete RPMI and centrifuged again at 350RCF. For analysis of cytokine production cells were seeded into round bottom 96-well plates and stimulated with 100 ng/ml Phorbol 12-myristate 13-acetate (Sigma-Aldrich) and 1 µg/ml ionomycin (Sigma-Aldrich) in the presence of 1X Brefeldin A (eBioscience) for 4 h before being prepared for intracellular flow cytometry.

### Intracellular Flow Cytometry

Cells were washed twice and treated with CD16/32 (eBiosciences) for 15 min at 4°C before being surface stained for CD4 GK1.5 (PerCP or FITC), CD8 53-6.7 (FITC or APC), and CD25 PC61.5 (APC) (eBiosciences). Cells were prepared for intracellular staining using eBiosciences FoxP3 staining kit. Following fixation and permeabilization, the cells were treated with 2% rat serum to prevent non-specific binding. Cells were stained for FOXP3 FJK-16s (PE), IFN-γ XMG1.2 (PE), and Ki67 SolA15 (PE) (eBiosciences) for 30 min and acquired using an Accuri C6 flow cytometer (BD Biosciences).

### Cryo Imaging

For imaging of organs, mice were sacrificed 48 h following MAPC cells administration by cervical dislocation. Organs of interest were harvested and put onto a thin layer of black OCT (Bioinvision Inc., Cleveland, OH, USA) in peel away molds kept on ice. Organs were covered in OCT and frozen on a metal block chilled in liquid nitrogen. Once OCT had solidified, the samples were transferred to −80°C. Sectioning and imaging were carried out using the automated CryoViz™ imaging system (BioInvision). Images were then processed to generate 3D images using CryoViz™ processing, and the number of detected cells was quantified using cell detection software (BioInvision). For whole mouse imaging, mice were humanely sacrificed using an IP injection of pentobarbitone. OCT was rubbed into the carcass, against the direction of fur to minimize air bubbles. The carcass was put onto a thin layer of OCT in a boat made from heavy duty aluminum foil with the ventral side facing down. Extra OCT was poured over the carcass to cover the mouse. Heavy duty tin foil was wrapped around the aluminum boat, and the sample was put into a box containing liquid Nitrogen. Once OCT had solidified, the samples were transferred to −80°C.

### Ethics

All procedures involving animals were carried out by licensed personnel. Ethical approval for all work was received from the research ethics committee of Maynooth University (under approval number BSRESC-2017-011). All procedures were carried out under an approved license from the Health Product Regulatory Authority.

### Statistics

Statistical analysis was performed using GraphPad Prism software (GraphPad, San Diego, CA, USA, http://www.graphpad.com/company). Statistical significance between experimental groups was tested using a Mann–Whitney test. Data are presented as the ±SEM. *p*-Values of *p* < 0.05 (*), *p* < 0.01 (**), and *p* < 0.001 (***) were considered statistically significant.

## Results

### MAPC Suppress IL-7-Driven Activation of T Cells *In Vivo*

**Figure 1:**
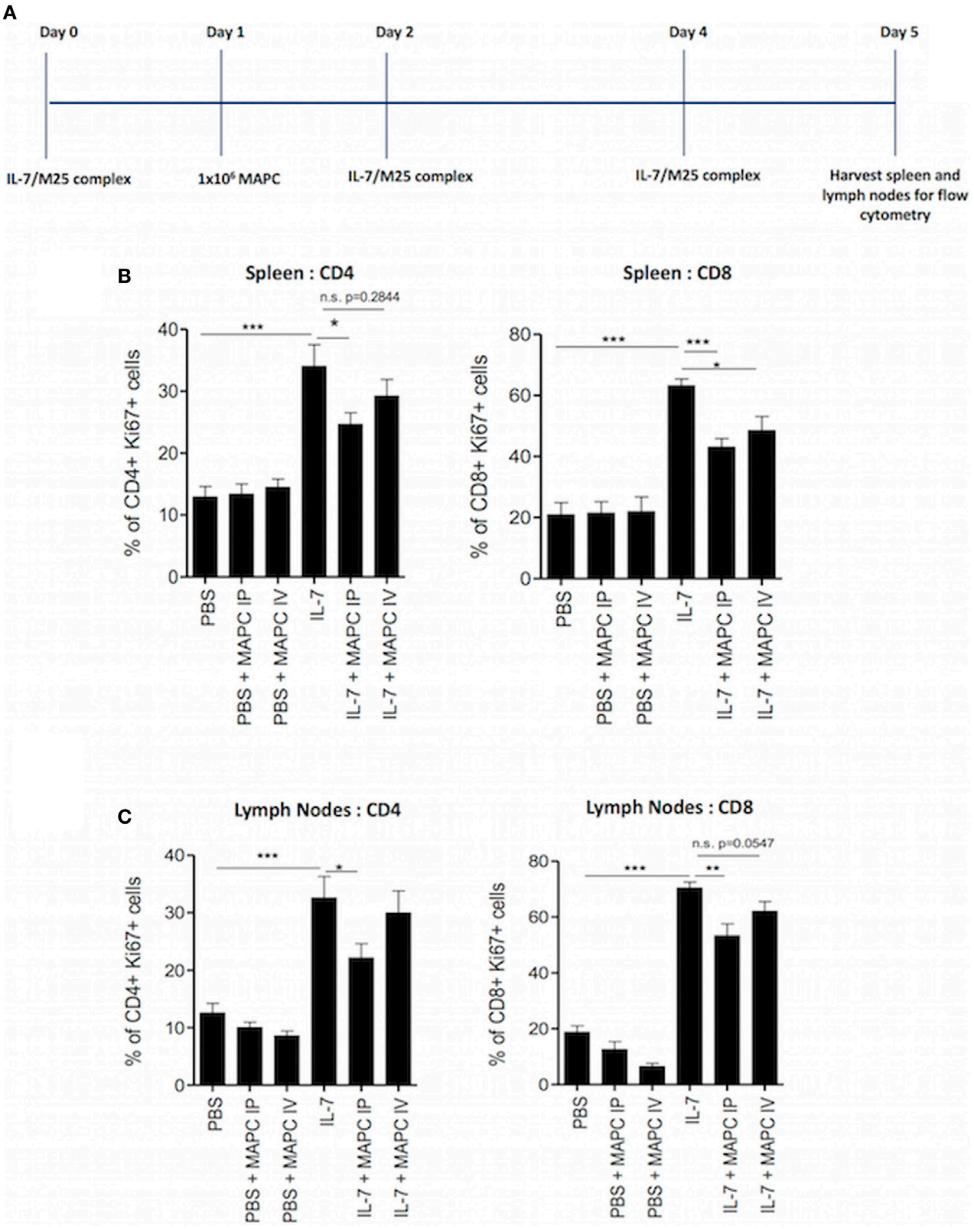
Multipotent adult progenitor cells (MAPC) suppress IL-7-driven proliferation of T cells *in vivo*. **(A)** Schematic timeline of IL-7-driven homeostatic proliferation experiments. Recombinant IL-7 conjugated to M25 or PBS was administered intraperitoneally (IP) on days 0, 2, and 4. 1 × 10^6^ MAPC cells were administered IP or intravenously (IV) on day 1. Lymph nodes and spleens were harvested on day 5. **(B)** Bar graphs demonstrating that both MAPC IV and MAPC IP reduce the frequency of Ki67^+^ CD4^+^ and CD8^+^ cells in the spleen (PBS: *n* = 13, PBS + MAPC IP: *n* = 17, PBS + MAPC IV: *n* = 13, IL-7: *n* = 12, IL-7 + MAPC IP: *n* = 17, and IL-7 + MAPC IV: *n* = 13) **(C)** Bar graphs demonstrating that MAPC IP but not MAPC IV reduce the frequency of Ki67^+^ CD4^+^ and CD8^+^ T cells in the lymph nodes (PBS: *n* = 12, PBS + MAPC IP: *n* = 15, PBS + MAPC IV: *n* = 13, IL-7: *n* = 12, IL-7 + MAPC IP: *n* = 17, and IL-7 + MAPC IV: *n* = 13). Results are indicative of three independent experiments using three MAPC donors *p < 0.05, **p < 0.01, and ***p < 0.001.

It is well established that IL-7 is an important stimulus for the homeostatic expansion of T cells, and we have previously shown that MAPC cells suppress the activation of T cells in response to IL-7 *in vitro* (28). Therefore, we hypothesized that MAPC cells would suppress IL-7-driven stimulation of T cells *in vivo*. To study the effects of MAPC cells on the homeostatic expansion of T cells *in vivo*, we used a system wherein recombinant IL-7 conjugated to anti-IL-7 or PBS was administered IP on days 0, 2, and 4. Human MAPC cells (1 × 10^6^) were administered either IV or IP on day 1. Spleens and lymph nodes were then harvested and processed for flow cytometry on day 5 (Figure 1A). Using Ki67 as a marker of proliferation, we used intracellular flow cytometry to investigate the effects of MAPC on IL-7-driven proliferation of CD4^+^ and CD8^+^ T cells. MAPC cells administered to PBS groups were used as an internal control, and neither MAPC cells administered IP or IV affected the proliferation of CD4^+^ or CD8^+^ cells in either the spleen or lymph nodes (Figure 1). As expected, IL-7 administration significantly increased the frequency of Ki67 expressing CD4^+^ and CD8^+^ T cells in both the spleen and lymph nodes (Figure 1). In the spleen, MAPC cells (from three different donors) administered both IV and IP reduced the frequency of Ki67^+^ CD4^+^ and Ki67^+^ CD8^+^ T cells (Figure 1B). Notably, only MAPC cells administered IP significantly reduced the frequency of both Ki67^+^CD4^+^ and Ki67^+^CD8^+^ T cells. While MAPC IV significantly reduced the frequency of Ki67^+^ CD8^+^ T cells, there was no significant difference (*p* = 0.2844) in the frequency of Ki67^+^ CD4^+^ T cells in the IL-7 + MAPC IV group (Figure 1B). Interestingly, in the lymph nodes MAPC IP but not MAPC IV reduced Ki67 expression by both CD4^+^ (*p* ≤ 0.05) and CD8^+^ T cells (*p* ≤ 0.01) (Figure 1C).

**Figure 2:**
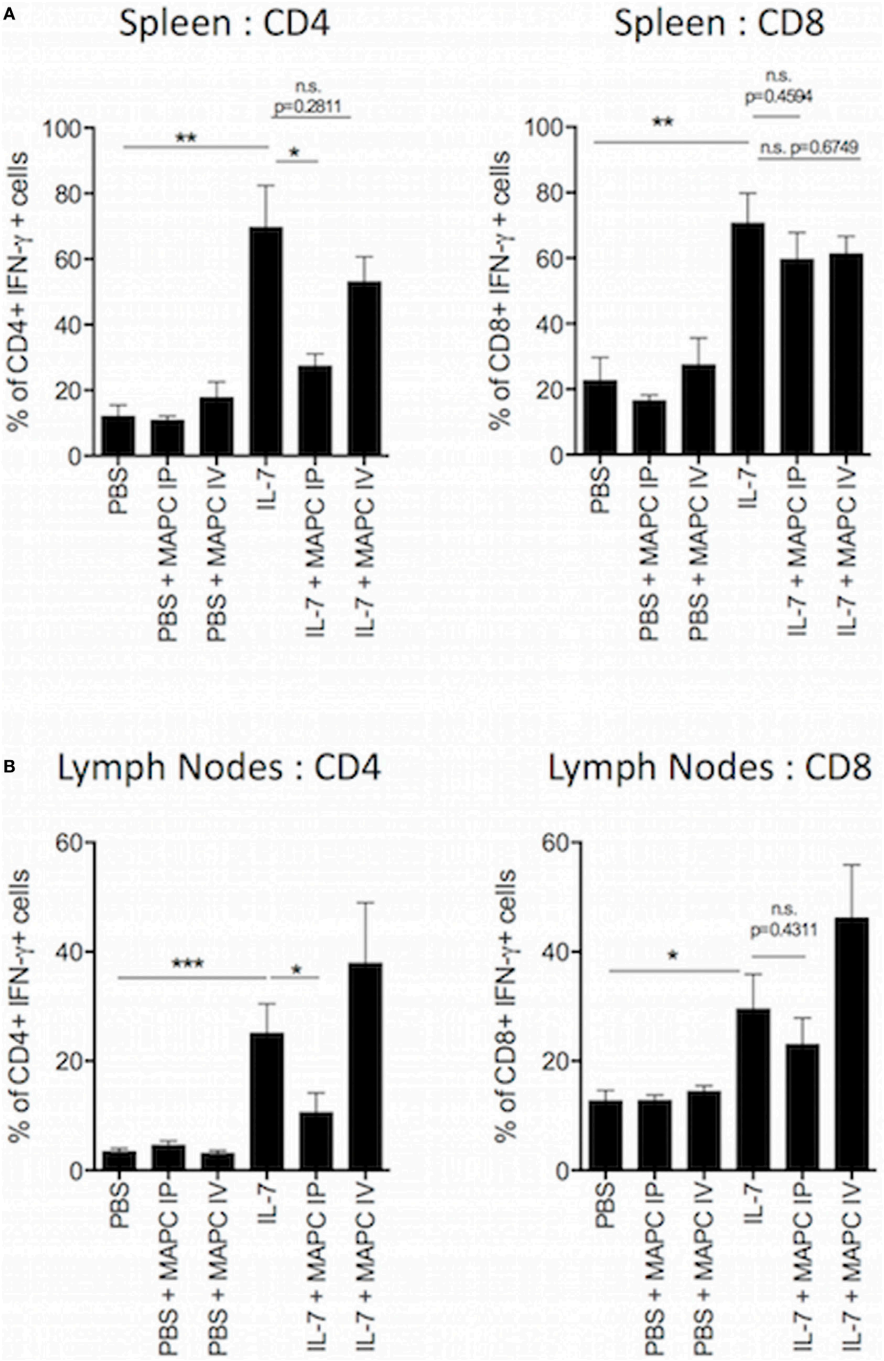
Multipotent adult progenitor cells (MAPC) suppress IL-7-induced interferon-γ (IFN-γ) production by T cells *in vivo*. Recombinant IL-7 conjugated to M25 or PBS was administered intraperitoneally (IP) on days 0, 2, and 4. 1 × 10^6^ MAPC were administered IP or intravenously (IV) on day 1. Lymph nodes and spleens were harvested on day 5. **(A)** The frequency of IFN-γ producing CD4^+^ and CD8^+^ in the spleen was significantly increased following IL-7 administration. Both MAPC IP and MAPC IV reduced IFN-γ production by T cells (PBS: *n* = 8, PBS + MAPC IP: *n* = 12, PBS + MAPC IV: *n* = 8, IL-7: *n* = 6, IL-7 + MAPC IP: *n* = 11, IL-7 + MAPC IV: *n* = 8). **(B)** Bar graphs demonstrating that MAPC IP but not MAPC IV reduce the frequency of IFN-γ production by CD4^+^ and CD8^+^ T cells in the lymph nodes (PBS: *n* = 8, PBS + MAPC IP: *n* = 11, PBS + MAPC IV: *n* = 7, IL-7: *n* = 7, IL-7 + MAPC IP: *n* = 10, and IL-7 + MAPC IV: *n* = 7). Results are indicative of two independent experiments using two MAPC donors. *p < 0.05, **p < 0.01, and ***p < 0.001.

The homeostatic expansion of T cells is known to promote the expansion of pro-inflammatory T cells and skew the immune compartment toward a Th1-like population. These Th1 cytokines are known to contribute to allograft rejection (7) and promote GvHD (42). Based on our previous data, we hypothesized that MAPC cells would suppress the production of pro-inflammatory cytokines by T cells in response to IL-7. Therefore, we examined the production of interferon-γ (IFN-γ) by T cells following *ex vivo* culture with PMA, ionomycin, and brefeldin A for 4 h. Administration of MAPC cells to PBS groups had no effect on the production of IFN-γ by T cells (Figure 2). As expected, IL-7 administration increased the frequency (Figure 2) and MFI (Figure S1 in Supplementary Material) of CD4^+^ and CD8^+^ T cells producing IFN-γ in both the spleen and the lymph nodes. In the spleen this effect was reduced when MAPC cells were administered both IV and IP (Figure 2A; Figure S1 in Supplementary Material). Similar to the proliferation data, only MAPC IP suppressed this in the lymph nodes, with MAPC IV having no effect at this site (Figure 2B; Figure S1 in Supplementary Material).

### MAPC Do Not Suppress T Cell Proliferation Following Lymphodepletion

**Figure 3:**
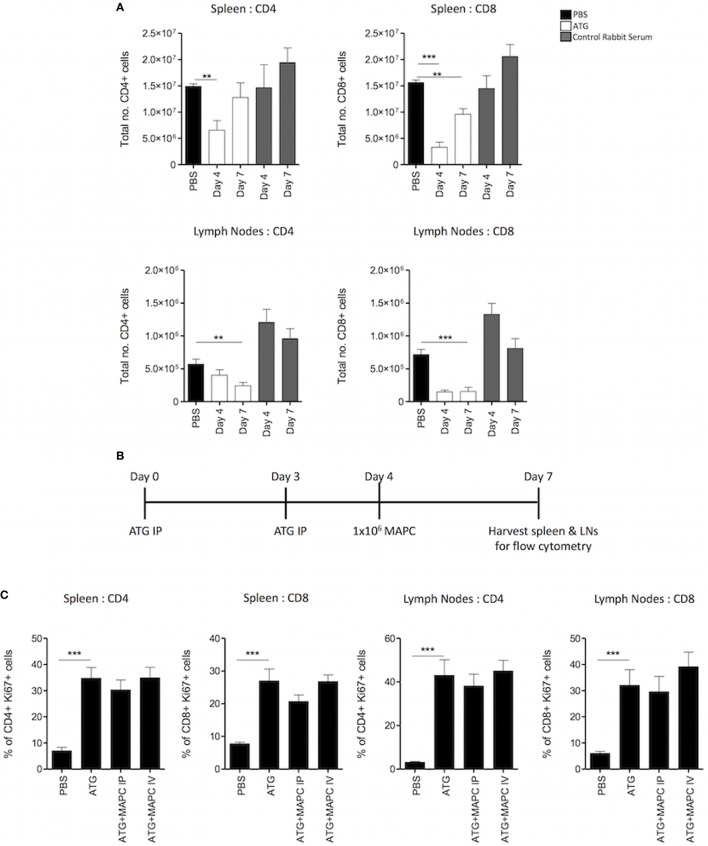
Multipotent adult progenitor cells (MAPC) have no effect on T cell proliferation following lymphodepletion. 50 mg/kg anti-thymocyte globulin (ATG) was administered intraperitoneally (IP) on days 0 and 3. **(A)** Spleens and lymph nodes were harvested on days 4 and 7, and total numbers of CD4^+^ and CD8^+^ cells were quantified using counting beads and flow cytometry. **(B)** Schematic timeline of experiments with MAPC (1 × 10^6^) administration [IP or intravenously (IV)] to the ATG model on day 4. **(C)** MAPC administered IV or IP had no effect on the proliferation of either CD4^+^ or CD8^+^ T cells in the spleens and lymph nodes following ATG administration (spleens; PBS: *n* = 10, ATG: *n* = 10, ATG + MAPC IP: *n* = 10, and ATG + MAPC IV: *n* = 8 and lymph nodes; PBS: *n* = 8, ATG: *n* = 10, ATG + MAPC IP: *n* = 9, and ATG + MAPC IV: *n* = 10). Results are indicative of two independent experiments using two MAPC donors. * < 0.05, **p < 0.01, and ***p < 0.001.

In the clinic, homeostatic proliferative (HP) occurs following T cell depletion due to the increase in the exposure of the remaining T cell pool to homeostatic stimuli. Therefore, in order to understand the effects of MAPC cells in a more translationally relevant setting, a lymphopenia-induced model of HP was set up using ATG to deplete T cells. The model developed by Ruzek et al. (43) was used as a guide for the dosing pattern of administering ATG IP on days 0 and 3, followed by harvest of spleen and lymph nodes on day 7 (43). Two doses of ATG were tested, with each mouse given either 25 or 50 mg/kg on days 0 and 3. Preliminary experiments demonstrated that 50 mg/kg ATG administered IP was required to deplete both CD4^+^ and CD8^+^ T cells in the spleen and lymph nodes optimally (Figure S2 in Supplementary Material). Normal rabbit serum was used as a control to confirm that depletive antibodies within the ATG were causing the reduction in T cell numbers. Administration of control serum did not deplete T cells but resulted in an increase in T cell numbers, presumably due to a xenogeneic T cell response (Figure S2 in Supplementary Material; Figure 3A).

To determine the kinetics of T cell depletion and HP, the number of T cells in the spleen and lymph nodes on days 4 and 7 were enumerated using counting beads. In the spleen, the CD4^+^ compartment was depleted at day 4, but numbers had increased by day 7. Similarly, depletion of CD8^+^ T cells was more pronounced at day 4 than day 7. On the contrary, while the number of CD4^+^ T cells in the lymph nodes was reduced on day 4, this reduction was more robust on day 7. In the CD8^+^ compartment, depletion was robust at both days 4 and 7 (Figure 3A). This aligns with previous studies showing that ATG is superior at depleting CD8^+^ T cells compared to CD4^+^ T cells (44). Furthermore, depletion of the T cell compartment occurs faster in the spleen than the lymph nodes. Ki67 expression by T cells was examined to ensure that HP was occurring at day 7 (timeline: Figure 3B). In both the spleen and lymph nodes, the frequency of Ki67^+^ CD4^+^ and CD8^+^ T cells was significantly enhanced in the ATG group compared to PBS controls (Figure 3C).

Since depletion had occurred in most cases at day 4, human MAPC cells (1 × 10^6^) were administered IV or IP at this point with the hypothesis that MAPC cell therapy would suppress proliferation of the T cell compartment, hindering T cell reconstitution (Figure 3B). Thus, the frequency of Ki67 expressing CD4^+^ and CD8^+^ T cells in the spleen and lymph nodes was examined in ATG groups following MAPC cell therapy. However, MAPC cells failed to suppress proliferation of T cells following lymphodepletion (Figure 3C) when delivered at day 4.

### MAPC IP Promote Treg and Suppress IFN-γ Production by CD8^+^ T Cells Following ATG Administration

**Figure 4:**
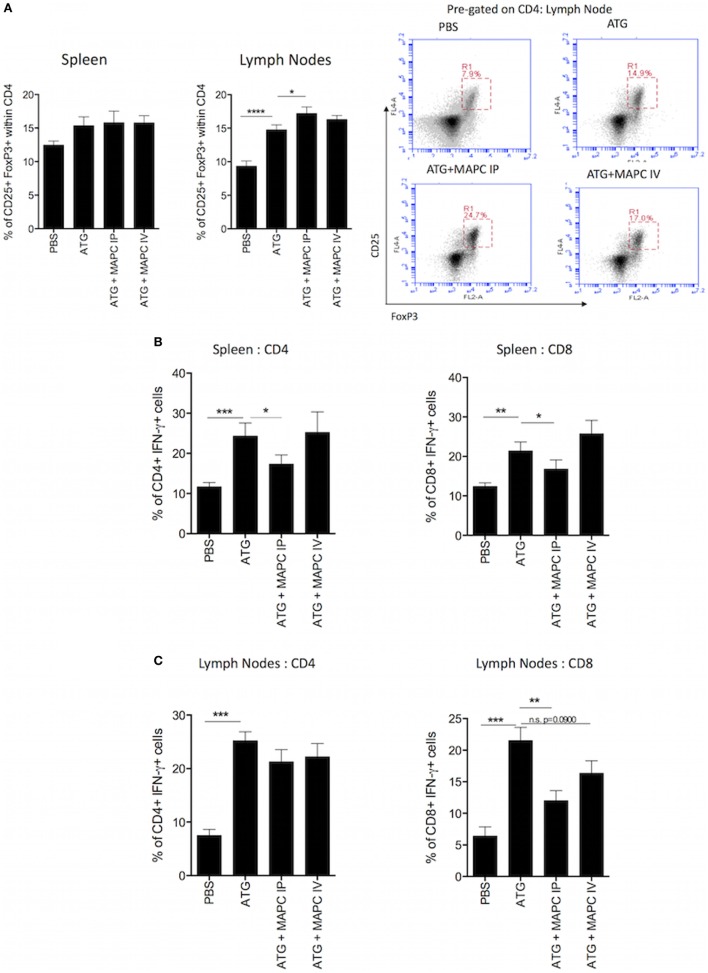
Multipotent adult progenitor cells (MAPC) promote Treg frequency and suppress interferon-γ (IFN-γ) production by T cells following lymphodepletion. **(A)** Bar graph and representative FACS plots demonstrate that anti-thymocyte globulin (ATG) increased the frequency of CD4^+^, CD25^+^, and FoxP3^+^ T cells in the spleen and lymph nodes, and MAPC cells administered intraperitoneally (MAPC IP) further increased this in the lymph nodes [PBS: *n* = 13, ATG: *n* = 13, ATG + MAPC IP: *n* = 11, and ATG + MAPC cells administered intravenously (MAPC IV): *n* = 13]. **(B)** MAPC IP reduced the frequency of IFN-γ producing CD4^+^ and CD8^+^ T cells in the spleen (PBS: *n* = 8, ATG: *n* = 10, ATG + MAPC IP: *n* = 10, and ATG + MAPC IV: *n* = 10). **(C)** MAPC IP reduced the frequency of IFN-γ producing CD8^+^ T cells but not CD4^+^ T cells in the lymph nodes (PBS: *n* = 12, ATG: *n* = 10, ATG + MAPC IP: *n* = 11, and ATG + MAPC IV: *n* = 10). Results are indicative of two independent experiments using two MAPC donors. *p < 0.05, **p < 0.01, and ***p < 0.001.

In the context of allograft rejection, the outcome of the immune reaction depends on the ratio of pro-inflammatory T cells to Tregs. The expansion of regulatory and memory T cells following ATG treatment is extensively reported (43–49). Therefore, we sought to examine if MAPC cell therapy promoted Treg while suppressing pro-inflammatory T cells. Splenocytes and lymph nodes were stained for CD4 and CD25 before being stained intracellularly for FoxP3. In the spleen, there was a slight increase in the frequency of CD25^+^, FoxP3^+^ cells within the CD4^+^ compartment following ATG administration and MAPC cell therapy had no further effect (Figure 4A). In the lymph nodes, ATG administration significantly increased the frequency of Treg. Importantly, MAPC cells further increased this population, with MAPC cells administered IP (*p* ≤ 0.05) being more effective than those administered IV (*p* = 0.0936) (Figure 4A).

Despite the fact that MAPC cells did not impair ATG-driven T cell HP, it was hypothesized that MAPC cells would suppress IFN-γ production by T cells in this lymphopenia-driven model in a similar manner to the IL-7 model. The frequency of IFN-γ^+^ cells was significantly increased in both the CD4^+^ and CD8^+^ compartments in the spleen and lymph nodes (Figures 4B,C) following lymphodepletion. MAPC IP significantly reduced the frequency of IFN-γ^+^ CD4^+^ and CD8^+^ splenocytes; however, MAPC IV had no such effect (Figure 4B). In the lymph nodes, MAPC cells had no effect on IFN-γ production by CD4^+^ cells; however, MAPC IP significantly reduced the percentage of IFN-γ producing cells within the CD8^+^ T cell population (*p* = 0.0027). MAPC IV also slightly reduced the frequency of IFN-γ^+^ CD8^+^ T cells in the lymph nodes; however, this reduction was not statistically significant (*p* = 0.0900) (Figure 4C).

### MAPC Cells Administered IP Do Not Gain Access to the Spleen

The vast majority of MSC and MAPC are trapped in the lung immediately following IV administration when administered to healthy animals, with only a small percentage of cells reaching distal organs such as the spleen (50). The biodistribution of MSC and MAPC cells administered IP has not been studied to such an extent. Thus, we sought to compare the distribution patterns of MAPC IP to MAPC IV to understand the differences in the efficacy of MAPC cells depending on their route of administration. MAPC cells were fluorescently labeled prior to administration, and whole mice, spleens and lymph nodes were harvested for cryo-imaging 48 h later. We hypothesized that MAPC cells administered IP would gain access to the lymph nodes and spleen which would explain their ability to suppress HP of T cells at this site.

**Figure 5:**
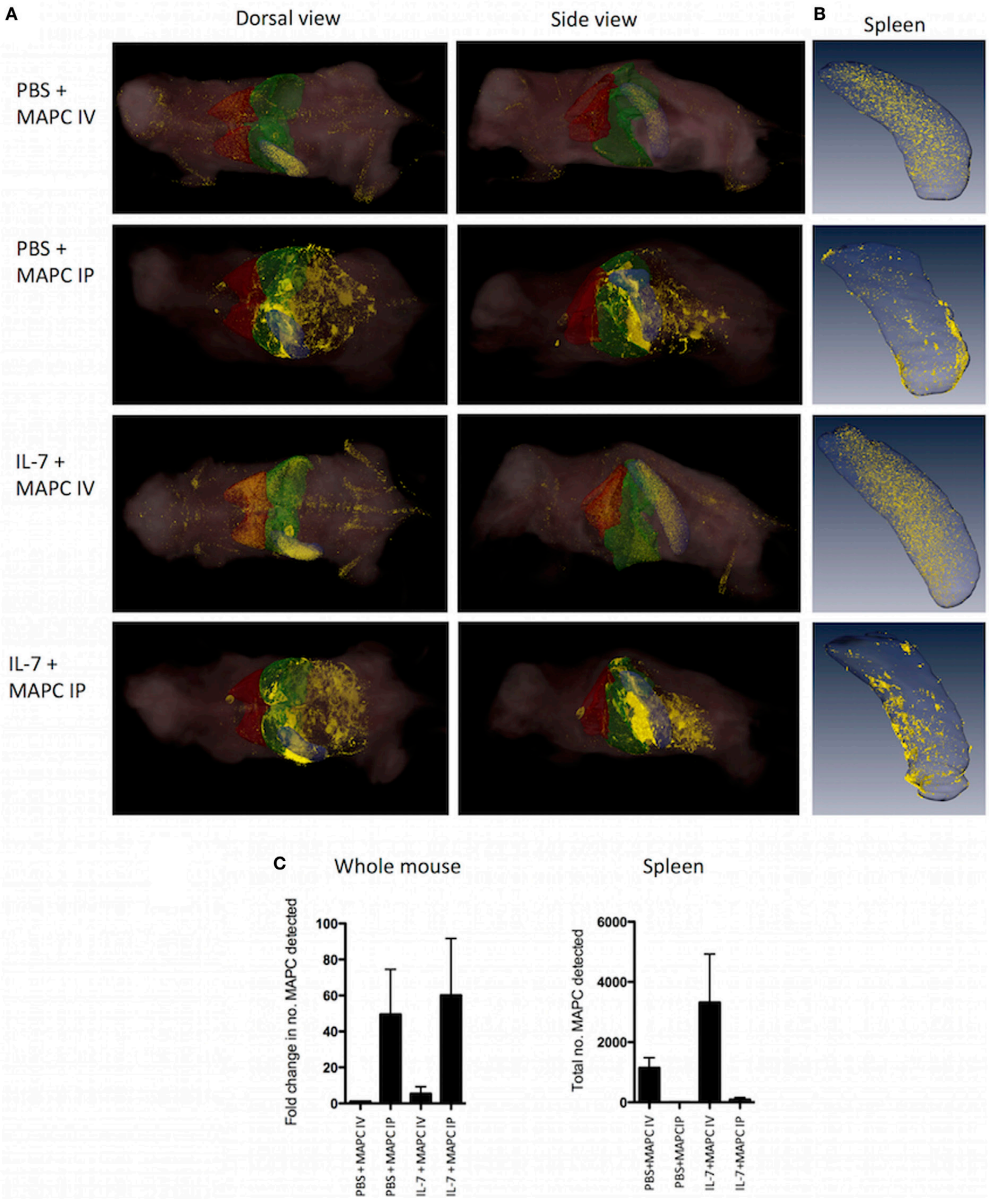
Multipotent adult progenitor cells (MAPC) administered intraperitoneally (IP) do not gain access to the spleen. 1 × 10^6^ Qtracker^®^ 625-labeled MAPC were administered [intravenously (IV) or IP] to PBS and IL-7-treated mice and whole mice (*n* = 3 per group) or spleen (*n* = 3 per group) were harvested 48 h later. **(A)** CryoViz Images (left: dorsal view, right: side view. The lungs are depicted in red, liver in green, and spleen in blue). The majority of cells administered IV were detected in the lung, liver, and spleen, while MAPC administered IP were found in the peritoneal area surrounding abdominal organs. Representative images present 3D analysis of MAPC-treated mice with detected MAPC shown in yellow. **(B)** CryoViz Images of the spleen. MAPC IV were detected in the spleen; however, MAPC IP did not gain access to the spleen, but were detected in the omental tissue surrounding the spleen (*n* = 3). 3D images show representative spleens with detected MAPC shown in yellow. **(C)** The fold change in the number of MAPC detected in whole mice and the total number of MAPC detected in spleens of mice at 48 h was quantified using quantification software (*n* = 3). For the fold change calculation, the number of MAPC detected in the PBS + MAPC IV group was set to 1, and the fold change in the number of MAPC detected in the other groups was calculated based on this.

Following IV administration to either the PBS or IL-7 mouse, the majority of these cells were detected in the lung, liver, and spleen. More MAPC cells were detected in the IL-7 group following IV administration of MAPC cells (Figures 5A,C). Differentially, MAPC IP were detected in the peritoneal area surrounding abdominal organs and appeared to be in clusters, which aligns well with the observations previously made by other groups (51, 52). Greater numbers of MAPC cells were detected in the PBS and IL-7 mice administered MAPC IP as shown by increase in fold change detected (Figure 5C). Next, the distribution of MAPC cells to the spleen of IL-7-treated mice was examined. Interestingly, while MAPC administered IV were detected within the spleen, MAPC IP were detected mostly in the omental tissue surrounding the spleen and did not gain access to splenic tissue (Figures 5B,C). Therefore, the differential effects of MAPC cells on various immune compartments in the spleen may be due to the contrast in persistence of MAPC IV compared to MAPC IP or it may be due to their distinct locations *in vivo*.

### MAPC Cells Require PGE2 to Suppress IFN-γ Production by CD8^+^ T Cells

**Figure 6:**
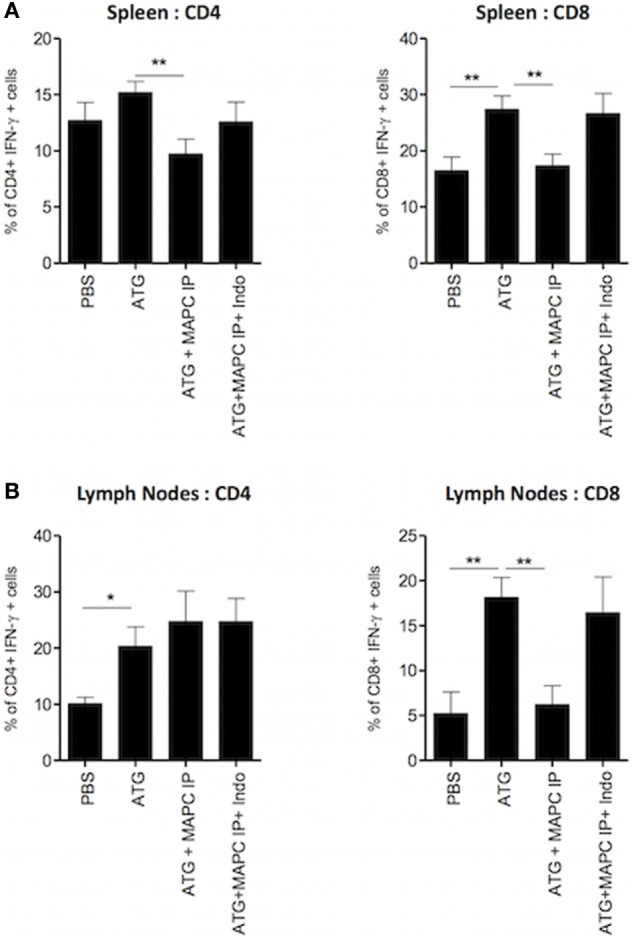
Multipotent adult progenitor cells (MAPC) administered intraperitoneally (IP) require prostaglandin E2 (PGE2) to suppress interferon-γ (IFN-γ) production by T cells. 100 mg/kg anti-thymocyte globulin (ATG) or control serum was administered over two doses given on days 0 and 3, followed by the administration of MAPC IP on day 4, and indomethacin (Indo) on days 4, 5, and 6. Spleens and lymph nodes were harvested on day 7 and examined for the production of IFN-γ by T cells. Bar graphs show that suppression of IFN-γ production by CD4^+^ and CD8^+^ T cells in the **(A)** spleen [PBS: *n* = 7, ATG: *n* = 7, ATG + MAPC IP: *n* = 9, and ATG + MAPC intravenous (IV): *n* = 7] and **(B)** lymph nodes (PBS: *n* = 4, ATG: *n* = 5, ATG + MAPC IP: *n* = 5, and ATG + MAPC IV: *n* = 4) was inhibited when Indo was administered on days 4, 5, and 6 of the lymphodepletion model. Results are indicative of two independent experiments using two MAPC donors. *p < 0.05 and **p < 0.01.

Prostaglandin E2 has been shown to be an important contributor to MSC- and MAPC-mediated immunosuppression in a number of *in vitro* and *in vivo* settings (24, 28, 30, 32). Furthermore, we have previously shown that suppression of IL-7-activated T cells by MAPC *in vitro* requires PGE2 (28). Thus, we hypothesized that the suppression observed by MAPC cells *in vivo* would be mediated by the same mode of action. To test this hypothesis, the cyclooxygenase 2 inhibitor indomethacin was administered to the ATG model along with MAPC IP on day 4. Indomethacin was injected again on days 5 and 6, followed by harvest of the spleens and lymph nodes on day 7. Indomethacin injected alone to ATG mice had no effect on IFN-γ production by T cells (Figure S3 in Supplementary Material). As expected, the frequency of IFN-γ-producing CD4^+^ and CD8^+^ cells in both the spleen (Figure 6A) and lymph nodes (Figure 6B) of the ATG group was increased compared to the PBS group. MAPC cells administered IP significantly suppressed both IFN-γ-producing CD4^+^ and CD8^+^ cells in the spleen, but only IFN-γ-producing CD8^+^ T cells in the lymph node. Administration of indomethacin reversed the effects of MAPC cells IP in this model, demonstrating that PGE2 is required for immunosuppressive effects of MAPC cells in this model (Figures 6A,B).

## Discussion

T cell-depletive therapies are commonly used in the clinic to prevent or delay allograft rejection; however, the consequential development of a pro-inflammatory T cell pool following lymphodepletion has not been addressed. While maintenance immunosuppression is used to inhibit the proliferation of T cells following induction therapy, none of the therapies currently on the market target the IL-7 axis, despite its known role in the homeostatic expansion of T cells during lymphopenia. We have previously shown that human MAPC cells suppress IL-7-driven proliferation and activation of T cells *in vitro* (28), and so this study sought to build on that data, by translating the findings to an *in vivo*, translationally relevant setting.

Using two models of HP we have shown here for the first time that MAPC suppress proliferation and IFN-γ production by both CD4^+^ and CD8^+^ T cells in response to both IL-7 and lymphodepletion *in vivo*. While MSC administered IV have been shown to be therapeutic in a range of conditions, a number of studies have suggested that MAPC cells require local delivery to mediate suppressive effects (31, 32). We explored the effect of delivery route in this model by injecting MAPC cells either IV or IP, with the hypothesis that MAPC IP would demonstrate superior immunosuppressive effects. In the spleen, both MAPC IP and MAPC IV suppressed IL-7-driven T cell proliferation and IFN-γ production; however, only MAPC IP had this effect in the lymph nodes. We then used a lymphodepletion model to test the hypothesis that MAPC cells would suppress T cell proliferation and activation during LIP. Administration of ATG depleted T cells in the spleen and lymph nodes and induced proliferation of the T cell pool; however, neither MAPC IP nor IV suppressed the T cell proliferation when delivered at Day 4. Despite having no effect on T cell proliferation, MAPC IP increased the frequency of Treg in the lymph nodes of ATG-treated mice, while simultaneously suppressing IFN-γ production by T cells in both the spleen and lymph nodes. Interestingly, MAPC IV demonstrated no immunosuppressive effect in this model when administered on day 4. Although, it is surprising that MAPC IP did not suppress T cell proliferation in the lymphodepletion model, this may be associated with the number of MAPC cells required to modulate T cell proliferation or the timing of when the MAPC were delivered. For example, lower numbers of MAPC cells did not significantly reduce T cell proliferation *in vitro* in response to IL-7 stimulation (28) but maintained the capacity to suppress cytokine production. In the context of immune reconstitution following lymphodepletion the ideal therapy would suppress pathogenic cytokine responses while sparing T cell reconstitution.

Overall, MAPC IP demonstrated superior immunosuppressive capacities in both of these models of HP. Imaging experiments revealed that while MAPC IP did not gain access to the spleen or lymph nodes, they were retained in the omental tissue surrounding these tissues. On the contrary, some MAPC IV did gain access to the spleen; however, access to the spleen did not correlate with the therapeutic efficacy of MAPC cells, supporting the hypothesis that MAPC cells mediate their effects through soluble factors. We have previously shown *in vitro* that the effects of MAPC cells on IL-7 activation of T cells are dependent on the production of PGE2 by MAPC cells (28). Furthermore, suppression of GvHD by intrasplenically delivered MAPC has been shown to be PGE2 dependent (32). Similarly, inhibition of PGE2 *in vivo* reversed the effects of MAPC IP during lymphopenia in this study. PGE2 has a short half-life *in vivo* and acts in a local manner, thus it is possible that MAPC IP are more effective due to being in closer proximity to the sites of interest in this model than MAPC IV which predominantly accumulate in the lung. MAPC IP are in closer proximity to the harvested lymph nodes than MAPC IV, thus PGE2 produced by MAPC IP would be more likely to affect T cells within the lymph nodes compared to PGE2 produced by MAPC IV. Furthermore, the whole mouse data demonstrated that MAPC IP were detected in greater numbers in the IL-7 model than MAPC IV. Thus, the greater numbers of MAPC IP could be producing more PGE2 continuously than MAPC IV. Furthermore, the superior efficacy of MAPC IP compared to MAPC IV might be due to the activation state of MAPC at different sites. *In vitro*, MAPC cells require contact with monocytes to produce PGE2 (28). MAPC IP may be more likely to be activated by monocytes or other cell types in the omentum than MAPC IV would be in the lung.

While this study demonstrates that MAPC cells suppress proliferation and activation of T cells in response to IL-7 and lymphopenia over a short period of time, the effect of MAPC cells on long-term immune reconstitution and how it would impact graft survival remains to be shown. The models used herein are simplified compared to more complex models of transplantation, and so future studies will focus on HP in the context of SOT and GvHD. Moreover, humanized animal models will improve the translation of these findings to the clinic and allow for more applicable analysis of the effects of human MAPC cells on human immune cell activity. Nevertheless, this study highlights that the timing of dosing, route of administration, or dosing of cells requires careful consideration. Given the xenogeneic nature of the model; human MAPC cells in mouse models of HP, there are limitations to this study. Little is understood about how human MSC communicate with mouse cells and tissues. However, human MSC can mediate protective effects in mouse models and humanized mouse models of inflammatory lung disease (53) and GvHD (29). Using shRNA knock down, our studies have shown that human MSC mediate protective effects through production of human HGF (53). While this study and others show that MAPC cells require close proximity to the spleen for therapeutic efficacy in transplant models (31, 32), this may not be a feasible route of administration in the clinic. A recent study has demonstrated that MAPC improve islet graft survival when the two cell types are co-transplanted in a composite pellet (54). Given, the accumulation of IP administered MAPC cells in the omentum and the consequent superior efficacy of MAPC cells, this may be an appropriate site for administration of such cellular therapies particularly in the context of islet transplantation. Recently, the omentum has been investigated as a potential site for islet transplantation (55) and this is being tested in clinical trials (NCT02213003). Thus, cotransplantation of MAPC cells along with allografts at the time of transplantation may be an effective and more practical approach where systemic administration is not sufficient.

The interactions between MSC and MAPC cells with macrophages have also been an area of intense research recently, with many studies showing that MSC/MAPC skew macrophage populations toward an M2, IL-10-producing phenotype. Furthermore, a number of studies have shown that the promotion of Treg *in vivo* by MAPC cells and MSC is dependent on the presence of macrophages (25, 34, 56). IP-injected MSC have previously been shown to form aggregates with macrophages (51), therefore, the superior therapeutic efficacy of MAPC IP in this study may be due to the interaction of MAPC cells with macrophages at this site. On the other hand, our previous study demonstrated that MAPC cells require IL-1β stimulation from monocytes to produce PGE2 (28), thus macrophages in the omental tissue may provide these signals, inducing PGE2 production by MAPC administered IP.

While other studies have demonstrated therapeutic efficacy of MAPC cells in models of allotransplantation (31, 32), the effect of MAPC cells on HP has only been studied previously *in vitro* (28). This study provides the first demonstration that clinical grade MAPC cells modulate homeostatic T cell responses *in vivo*. These data suggest that MAPC cells may prevent allograft rejection in part by modulating the T cell pool or pathogenic cytokine production during HP through production of PGE2. Thus, this study contributes to existing literature highlighting the potential of MAPC cells as a method of inhibiting allograft rejection and supports the idea that MAPC cells may be useful in controlling immune dysregulation *via* suppression of T cell cytokine production while sparing T cell reconstitution in lymphodepleted patients.

## Ethics Statement

This research involved the use of animal models. Ethical approval for this study was granted by Maynooth University Research Ethics Committee and authorization to conduct the animal model experiments was granted by the Scientific animal protection unit of the Health Products Regulatory Agency in Ireland.

## Author Contributions

FC performed research, data analysis, study design, and wrote the manuscript; TIMT and JLR contributed to study design and data analysis; and SRS and AET provided reagents, contributed to study design and data analysis. JMC and JPMCMC performed experiments and analysed data. KE designed and supervised the study and wrote the manuscript. All authors approved the final manuscript.

## Conflict of Interest Statement

FC is a PhD student at Maynooth University funded by an Irish Research Council Enterprise Partnership Scholarship funded in part by ReGenesys Ltd., a wholly owned subsidiary of Athersys Inc. JLR is a post-doctoral research associate at King’s College London, partially funded by a sponsored, unrestricted research agreement from Athersys Inc. TIMT is a Senior lecturer and principal investigator at King’s College London, in receipt of an unrestricted research agreement from Athersys Inc. SRS and AET are employees of Athersys Inc, and AT is a shareholder in Athersys Inc. KE is a lecturer and principal investigator at Maynooth University in receipt of an unrestricted research agreement from Athersys Inc. All other authors declare that the research was conducted in the absence of any commercial or financial relationships that could be construed as a potential conflict of interest.

## Supplementary Material

Figure S1 Multipotent adult progenitor cells (MAPC) suppress IL-7-induced interferon-γ (IFN-γ) production by T cells *in vivo*. Recombinant IL-7 conjugated to M25 or PBS was administered intraperitoneal (IP) on days 0, 2, and 4. 1 × 10^6^ MAPC were administered IP or intravenous (IV) on day 1. Spleens **(A)** and lymph nodes **(B)** were harvested on day 5. Bar graphs demonstrating that both MAPC IV and MAPC IP reduce the MFI of IFN-γ levels produced by CD4^+^ and CD8^+^ cells in the spleen while only MAPC IP have this effect in the lymph nodes (PBS: *n* = 8, PBS + MAPC IP: *n* = 12 PBS + MAPC IV: *n* = 8, IL-7: *n* = 8, IL-7 + MAPC IP: *n* = 12, and IL-7 + MAPC IV: *n* = 8). Results are indicative of two independent experiments using two MAPC donors. **p* < 0.05, ***p* < 0.01, and ****p* < 0.001.

Figure S2 100 mg/kg anti-thymocyte globulin (ATG) administered intraperitoneal (IP) depletes T cells *in vivo*. 50 or 100 mg/kg ATG was administered on days 0 and 3 either IP or intravenous (IV). Spleens and lymph nodes were harvested on day 7, and the number of T cells was enumerated by flow cytometry. 100 mg/kg ATG administered IP was the optimal dose and route for T cell depletion in this setting (*n* = 3). **p* < 0.05, ***p* < 0.01, and ****p* < 0.001.

Figure S3 Indomethacin does not impair interferon-γ (IFN-γ) production by T cells following anti-thymocyte globulin (ATG) administration. ATG was administered on days 0 and 3 followed by intraperitoneal (IP) injection of 30 µg Indomethacin (Indo) on days 4, 5, and 6. Spleens were harvested on day 7 and examined for the production of IFN-γ. Indo had no effect on the production of IFN-γ by splenic CD8^+^ T cells (*n* = 5).
