# Supplementary figures and images for: Multipotent Adult Progenitor Cells Suppress T Cell Activation in In Vivo Models of Homeostatic Proliferation in a Prostaglandin E2-Dependent Manner
Source: Front Immunol. 2018 Apr 23;9:645. doi: 10.3389/fimmu.2018.00645 (PMC5925221; doi:10.3389/fimmu.2018.00645)

A

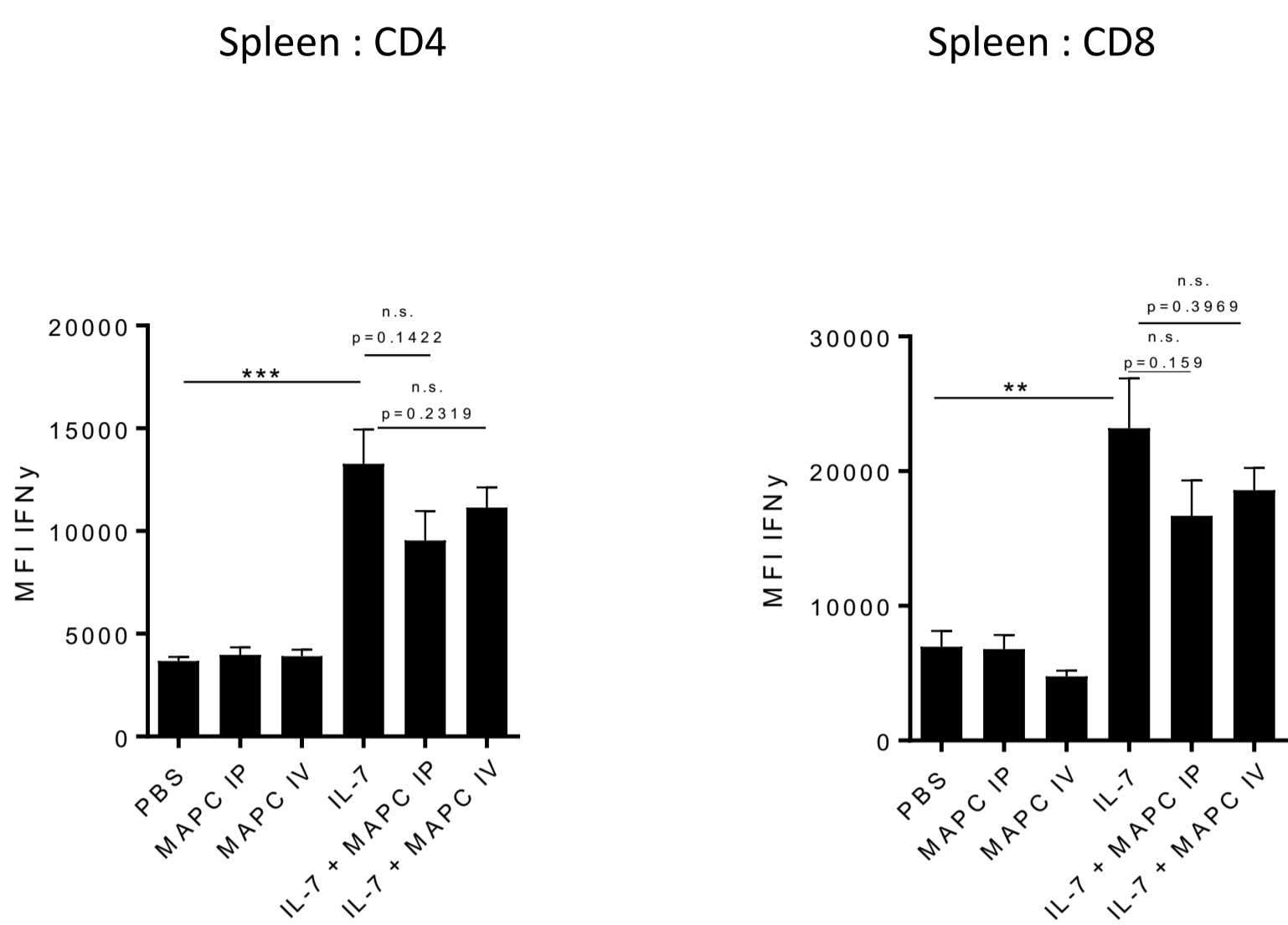

B

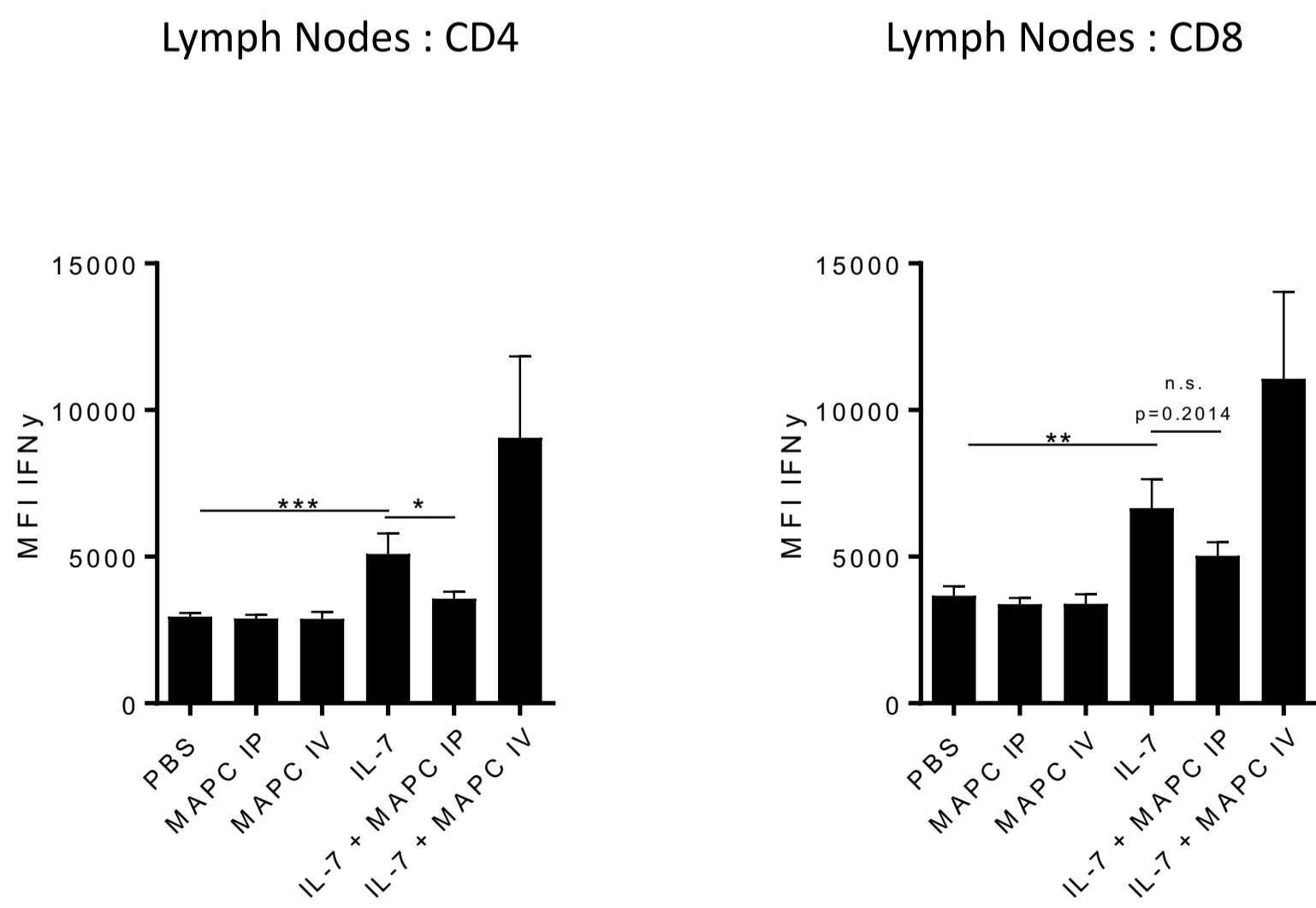

PBS  
ATG  
Control serum

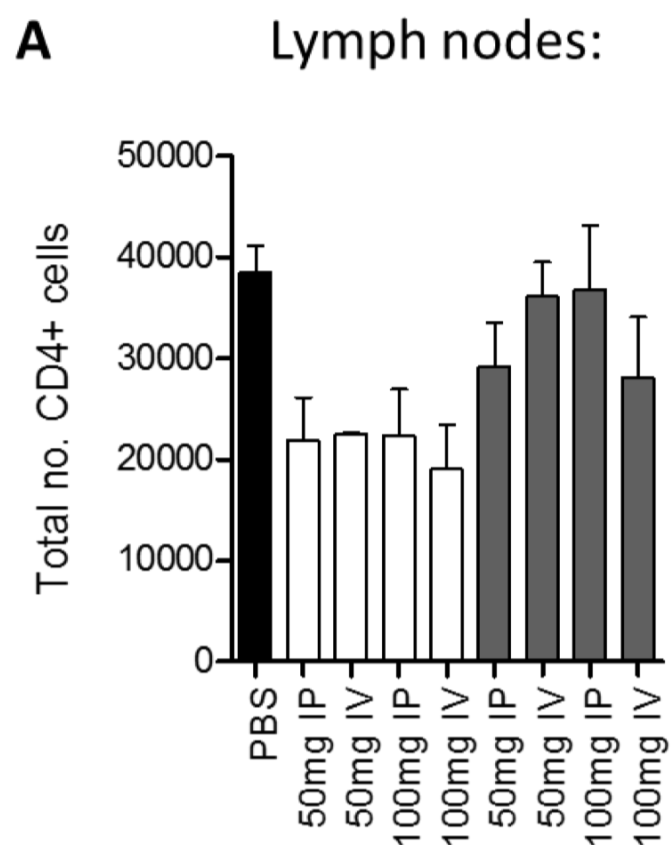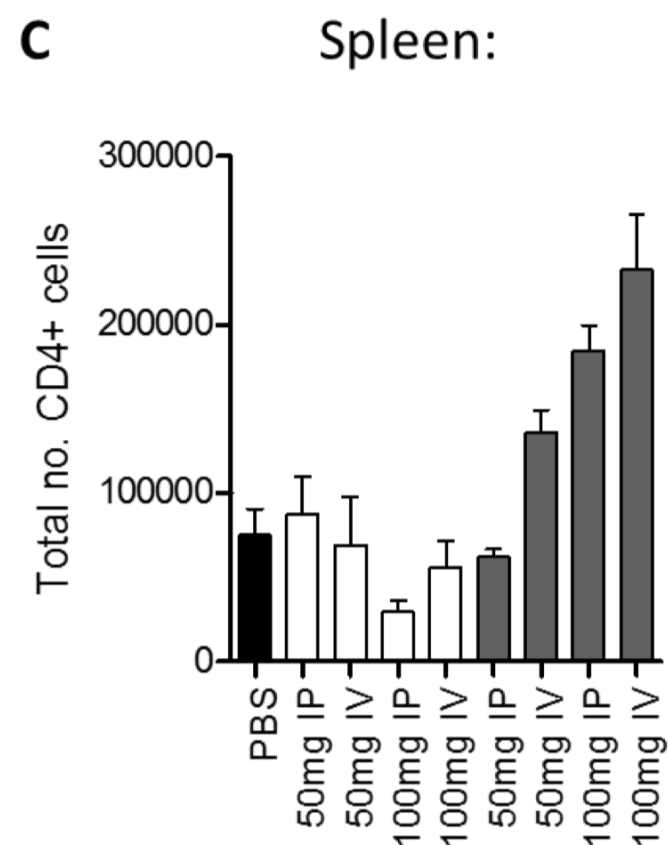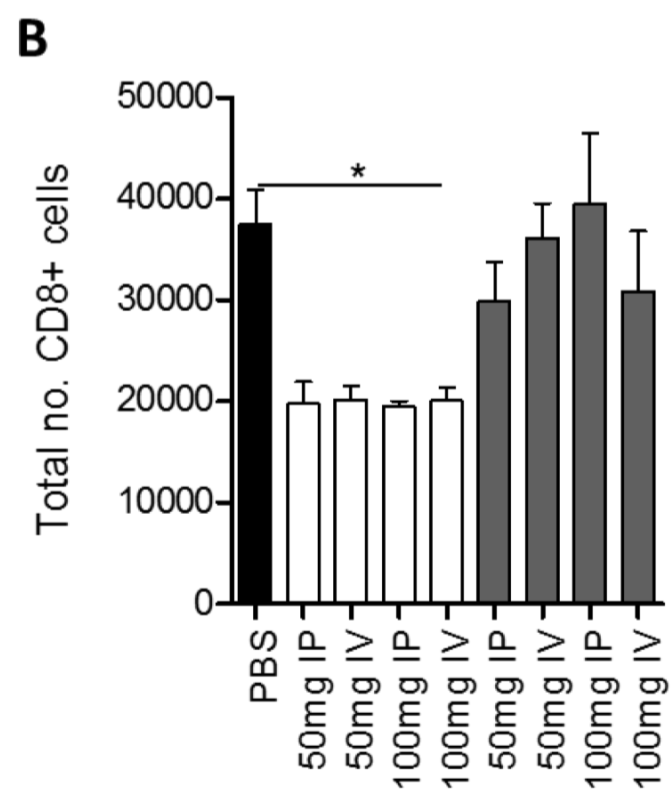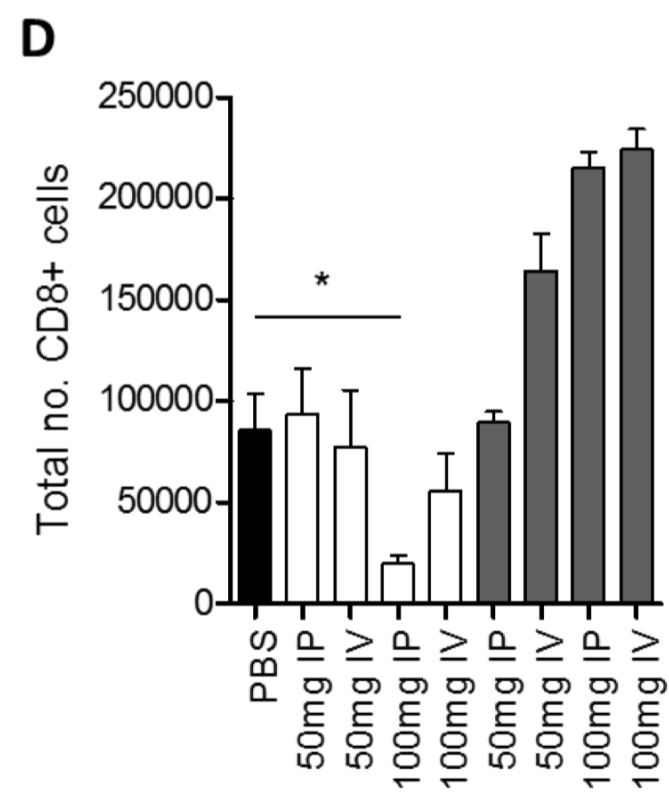

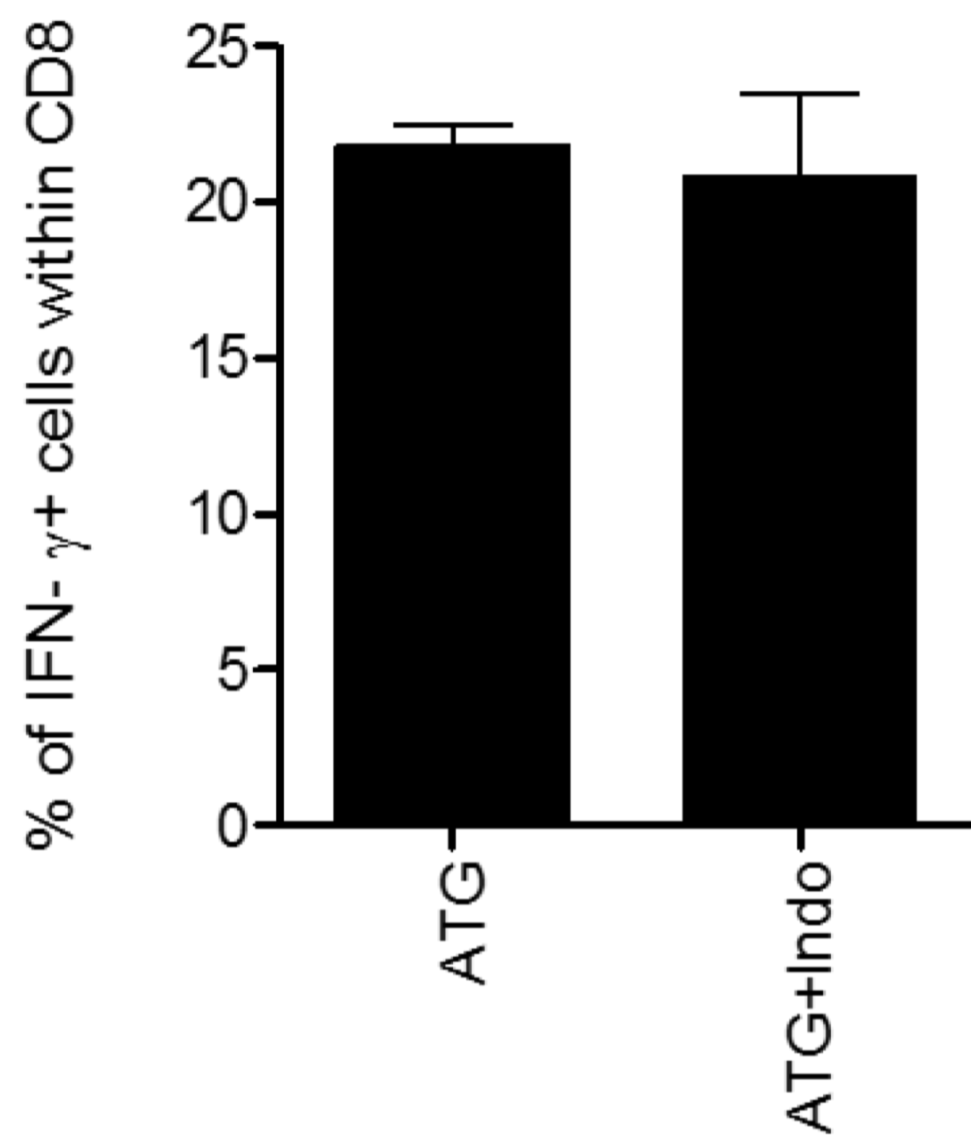

Supplement: Figure S1 — Multipotent adult progenitor cells (MAPC) suppress IL-7-induced interferon-γ (IFN-γ) production by T cells in vivo. Recombinant IL-7 conjugated to M25 or PBS was administered intraperitoneal (IP) on days 0, 2, and 4. 1 × 10^6^ MAPC were administered IP or intravenous (IV) on day 1. Spleens (A) and lymph nodes (B) were harvested on day 5. Bar graphs demonstrating that both MAPC IV and MAPC IP reduce the MFI of IFN-γ levels produced by CD4+ and CD8+ cells in the spleen while only MAPC IP have this effect in the lymph nodes (PBS: n = 8, PBS + MAPC IP: n = 12 PBS + MAPC IV: n = 8, IL-7: n = 8, IL-7 + MAPC IP: n = 12, and IL-7 + MAPC IV: n = 8). Results are indicative of two independent experiments using two MAPC donors. *p < 0.05, **p < 0.01, and ***p < 0.001. [file presentation_1.PDF]
